# Conformation and dynamics of the ligand shell of a water-soluble Au_102_ nanoparticle

**DOI:** 10.1038/ncomms10401

**Published:** 2016-01-21

**Authors:** Kirsi Salorinne, Sami Malola, O. Andrea Wong, Christopher D. Rithner, Xi Chen, Christopher J. Ackerson, Hannu Häkkinen

**Affiliations:** 1 Department of Chemistry, Nanoscience Center, University of Jyväskylä, FI-40014 Jyväskylä, Finland; 2 Department of Physics, Nanoscience Center, University of Jyväskylä, FI-40014 Jyväskylä, Finland; 3 Department of Chemistry, Colorado State University, Fort Collins, Colorado 80523, USA

## Abstract

Inorganic nanoparticles, stabilized by a passivating layer of organic molecules, form a versatile class of nanostructured materials with potential applications in material chemistry, nanoscale physics, nanomedicine and structural biology. While the structure of the nanoparticle core is often known to atomic precision, gaining precise structural and dynamical information on the organic layer poses a major challenge. Here we report a full assignment of ^1^H and ^13^C NMR shifts to all ligands of a water-soluble, atomically precise, 102-atom gold nanoparticle stabilized by 44 para-mercaptobenzoic acid ligands in solution, by using a combination of multidimensional NMR methods, density functional theory calculations and molecular dynamics simulations. Molecular dynamics simulations augment the data by giving information about the ligand disorder and visualization of possible distinct ligand conformations of the most dynamic ligands. The method demonstrated here opens a way to controllable strategies for functionalization of ligated nanoparticles for applications.

## Abstract (web summary)

The core structure of inorganic nanoparticles, stabilized by a passivating layer of organic molecules, is often known but information about the organic layer is tougher to derive. Here, the authors use NMR and computational methods to probe the ligand disorder and visualize possible ligand conformations.

## Graphical abstract

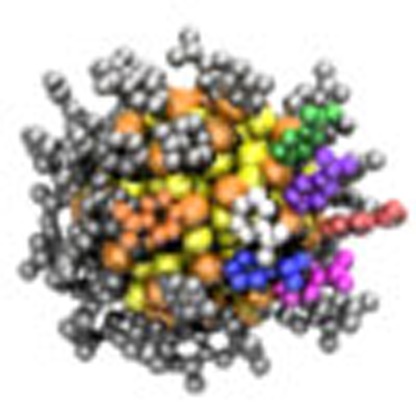

Inorganic nanoparticles, including metals, semiconductors and metal oxides, comprise a common set of structures exhibiting an inorganic core ‘passivated' by an organic shell^1^,^2^. Ligated inorganic nanoparticles currently provoke widespread fundamental interest in their structural, optical and magnetic properties, which differ fundamentally from bulk counterparts. These nanomaterials already find application in biology^3^, medicine, solar energy^4^ and display panels^5^. Despite their widespread study, the structure of inorganic nanoparticles, especially of their surfaces, is often still a matter of conjecture^6^. While the structure of the inorganic fraction of nanoparticles is straightforwardly determined by methods such as transmission electron microscopy and/or powder diffraction^7^,^8^, the nature of the ligand/metal interface is often obscure.

In special cases where synthesis is successfully refined to produce ‘atomically precise' nanoparticles that can form well-ordered crystals, single-crystal X-ray methods can result in total structure determination^9^,^10^,^11^. Single-crystal X-ray methods reveal that the surfaces of nanoparticles can be markedly rearranged by ligand interactions, often into surprisingly low-symmetry structures^12^. These single-crystal X-ray structures highlight the shortcomings of conventional transmission electron microscopy methods for determining nanoparticle structure.

Multidimensional nuclear magnetic resonance (NMR) spectroscopy can reveal a three-dimensional structure with fidelity comparable to single-crystal X-ray crystallography. Indeed, in determining the complex structures of proteins, the method is ideal for addressing the structures and dynamical interactions of proteins or protein fragments that are recalcitrant to crystallization^13^. Importantly, NMR provides a sensitive method to probe the structure of the ligand shell of ligand-passivated inorganic nanoparticles in solution phase, and it has indeed been recently applied to a few cases of atomically precise gold nanoclusters stabilized by thiolates^14^,^15^,^16^,^17^.

Single-crystal X-ray diffraction studies^9^ yielded the total structure of Au_102_(pMBA)_44_ in 2007, revealing a complex ligand layer with 22 symmetry-unique ligand environments in C_2_ overall symmetry (pMBA stands for para-mercapto benzoic acid). Since then, its electronic structure^18^,^19^,^20^, spectroscopic^21^ properties and chiral^22^ properties as well as the propensity for thiolate ligand exchange^12^ have been studied, and this nanoparticle has been functionalized for site-specific labelling of enteroviruses by utilizing covalent^23^ and weak^24^ interactions with viral capsid proteins. NMR spectroscopy has previously revealed the protein-like complexity of the Au_102_(pMBA)_44_ ligand shell^15^, yielding a broad one-dimensional (1D) ^1^H spectrum comprising several overlapping signals. Here data from multidimensional NMR spectroscopy (nuclear Overhauser effect spectroscopy (NOESY), rotating frame nuclear Overhauser effect spectroscopy (ROESY), total correlated spectroscopy (TOCSY) ^1^H NMR and heteronuclear single-quantum correlation spectroscopy (HSQC) ^1^H–^13^C correlations), combined with theoretical calculations for ^1^H and ^13^C NMR shifts based on the known crystal structure of Au_102_(pMBA)_44_, augmented by molecular dynamics (MD) simulations of the ligand dynamics in water, allowed for a full assignment of the 1D NMR signals to the 22 symmetry-unique ligands. The successful strategy disclosed here opens a new avenue for structural and dynamical studies of ligated inorganic nanoparticles in the solution phase, and is generalizable to both organic and aqueous media. Resolving the structure and dynamics of the ligand layer with molecular precision can lead to highly controllable and accurate methods for functionalization of the ligated nanoparticles for various applications where the essential chemistry between the nanoparticle and environment takes place in solution phase.

## Results

### Solid-state structure

**Figure 1:**
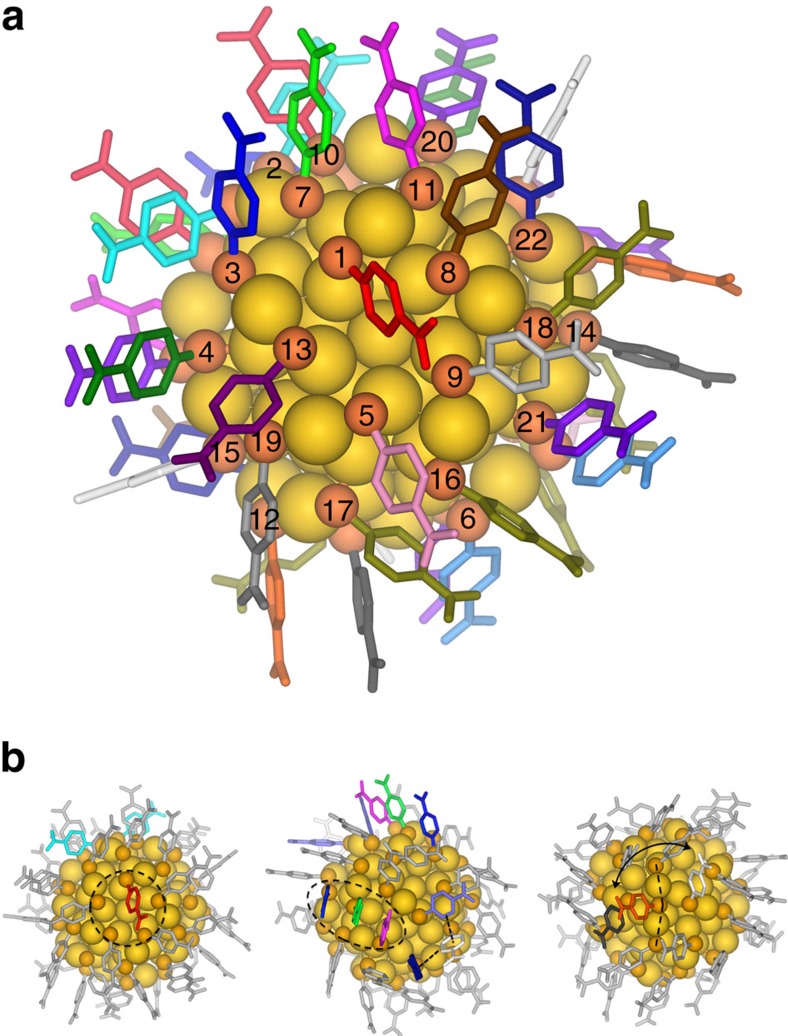
Crystal structure and selected special ligand environments of Au_102_(pMBA)_44_. (**a**) Total structure, with numbering and unique colouring of all the 22 symmetry-unique ligands. The same numbering scheme is used throughout this paper. Gold is yellow and sulfur is orange. (**b**) Selected special ligand environments on cluster surface: ligand-to-gold core (pMBA-1 and -2; red and cyan, respectively; left panel), face-to-face (pMBA-11, -7 and -3; magenta, green and blue, respectively; middle panel) and edge-to-face (pMBA-15, -22 and-20; white, navy and purple, respectively; middle panel) and dynamic ligands in the long gold–thiolate unit (pMBA-12, -15 and-14; orange, white and dark grey, respectively; right panel). For clarity, only the highlighted ligands have been coloured.

The atomic structure of Au_102_(pMBA)_44_ determined from the single-crystal X-ray experiment^9^ is shown in Fig. 1a. Within the C_2_ overall symmetry, the cluster contains 22 symmetry-unique pMBA ligands. The X-ray structure reveals that the pMBA ligands interact with the inorganic core through two types of gold–thiolate motifs—short RS-Au-SR and long RS-Au-(SR)-Au-SR units^9^. Although crystal packing has an effect on the ligand orientation due to the intermolecular interactions between neighbouring clusters in the solid state, the same ligand symmetry equivalence was previously suggested in the ^1^H NMR spectrum of Au_102_(pMBA)_44_ cluster in solution^15^. Therefore, an intrinsic ordering of the ligands on the cluster surface in solution may be similar to that observed in the solid state. The assignment of ligands to their symmetry-equivalent site on the cluster surface cannot be done solely based on the 1D proton spectrum due to high complexity of broad and overlapping signals. Thus, two-dimensional (2D) NMR techniques together with density functional theory (DFT) calculations and MD simulations were undertaken for full assignment of the symmetry-equivalent pMBA ligands in the 1D proton spectrum of Au_102_(pMBA)_44_ cluster.

### 2D NMR experiments and chemical shift assignment

**Figure 2:**
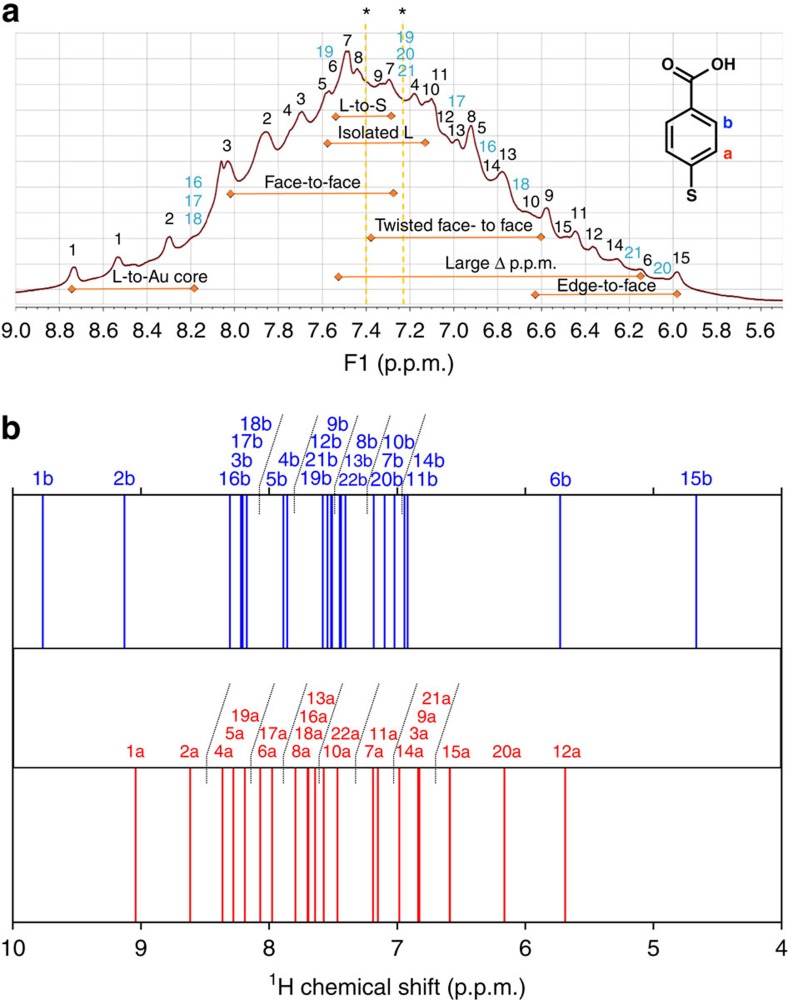
Ligand assignments in the proton NMR spectrum. (**a**) ^1^H NMR spectrum of Au_102_(pMBA)_44_ cluster in 0.3 M D_2_O–NaOH with full assignment of the 22 symmetry-equivalent pMBA ligands based on TOCSY spectrum. Strong correlation peaks have been marked with black and weaker signals in light blue (see TOCSY spectrum in Fig. 3). Special ligand environments affecting the chemical shift shielding have been identified in the spectrum (orange double arrows). Asterisk (*) denotes chemical shifts of free pMBA at 7.40 and 7.23 p.p.m. in 0.3 M D_2_O–NaOH (ref. 15). (**b**) DFT-calculated proton chemical shifts for H_a_ (red) and H_b_ (blue).

NMR spectroscopy is sensitive to the magnetic environment of atomic nuclei of the markers (such as ^1^H and ^13^C). The magnetic environment is modified by shielding, which is influenced by the electronegativity of neighbouring atoms, the interaction between markers and nearby metals, as well as orientation and distance of neighbouring functional groups. Owing to the restricted mobility of ligands bound at the cluster surface, the local magnetic environment becomes more pronounced as each of the symmetry-equivalent ligand will experience unique and distinguishable shielding based on their different local chemical environment on the cluster surface. Therefore, a set of 2D NMR experiments were recorded to resolve the individual chemical shifts of the 22 pMBA ligands in the 1D proton spectrum (Fig. 2a) and to elucidate neighbouring ligands and ligand order on the cluster surface.

pMBA has two chemically different protons (H_a_ closer to sulfur and H_b_ closer to COOH group; see inset to Fig. 2a), which give rise to two doublets in the proton spectrum. As seen in Fig. 2a, the 1D proton spectrum is extremely broad and peaked around the chemical shifts of free pMBA at 7.40 and 7.23 p.p.m. in the reference solvent. Figure 2b shows the theoretically calculated chemical shifts for H_a_ and H_b_ based on the structure of the ligand layer in the X-ray crystal data.

**Figure 3:**
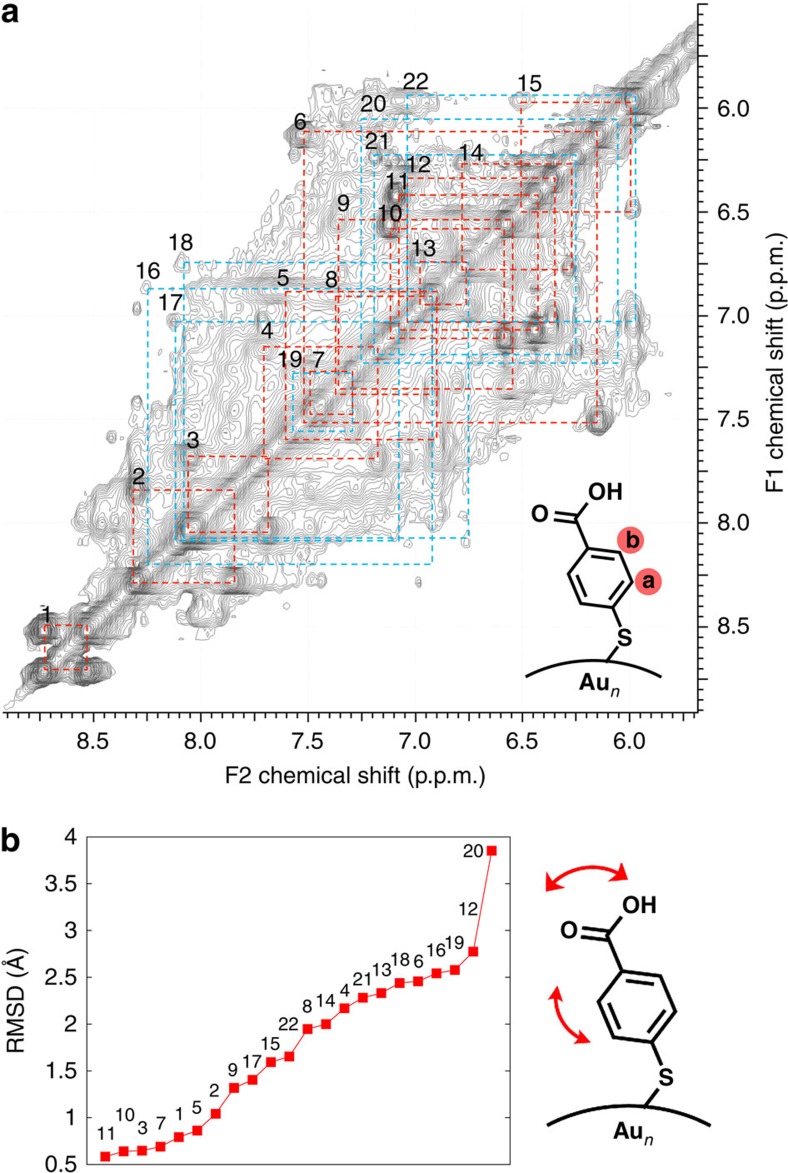
TOCSY data and dynamics of ligands. (**a**) TOCSY spectrum of Au_102_(pMBA)_44_ cluster in 0.3 M D_2_O–NaOH with full assignment of the 22 symmetry-equivalent pMBA ligands. Strong correlation peaks have been marked with red and weaker correlation peaks are marked with blue. (**b**) Root mean squared deviations (r.m.s.d.) of all the symmetry-unique 22 ligands in MD simulations of Au_102_(pMBA)_44_ in water at room temperature.

**Figure 4:**
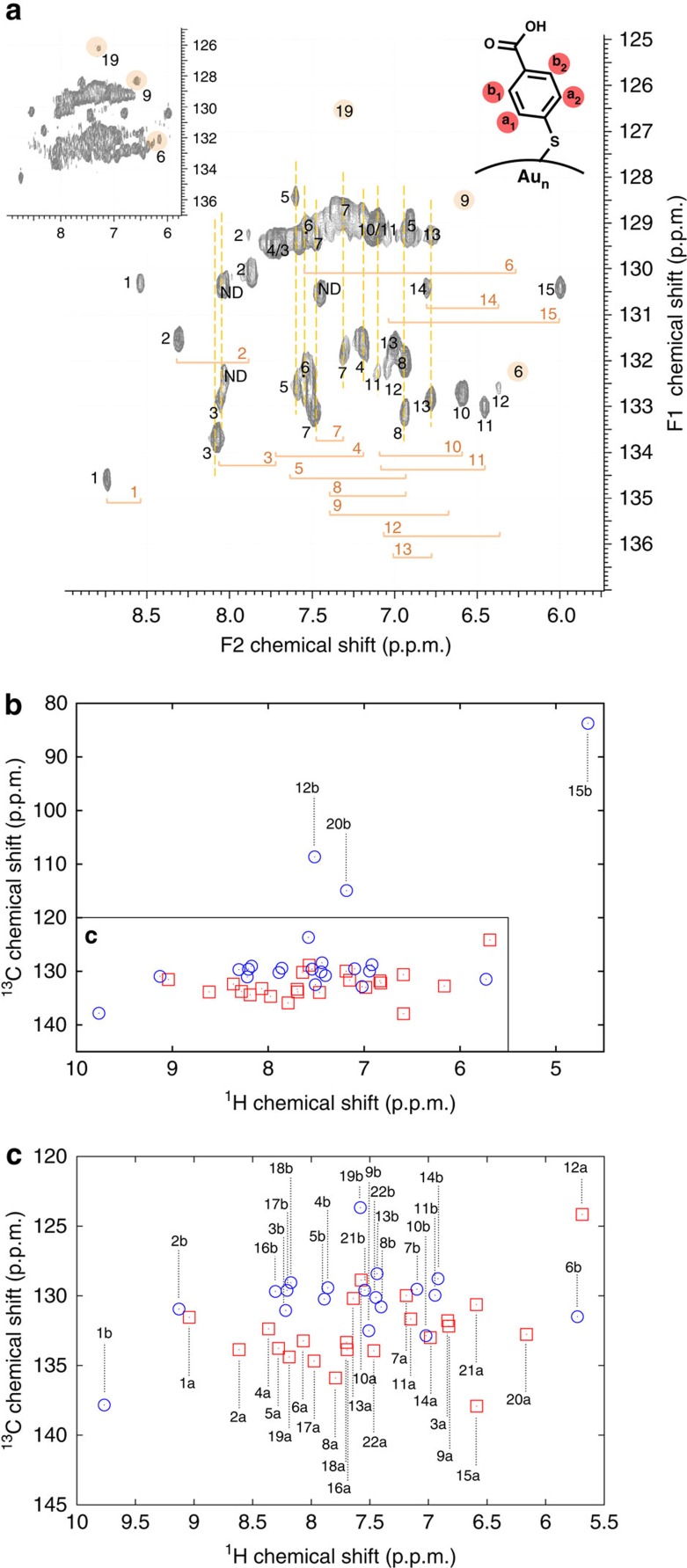
HSQC data. (**a**) HSQC spectrum of Au_102_(pMBA)_44_ cluster in 0.3 M D_2_O–NaOH showing assignment of ^1^H–^13^C correlation peaks. For some of the less-mobile ligands, correlation peaks for both a_1_ and a_2_ and/or b_1_ and b_2_ carbons could be identified. Inset shows the entire spectrum with higher signal-to-noise level and ND denotes ‘not determined'. (**b**,**c**) Calculated ^1^H–^13^C correlation chemical shifts. (**c**) The congested area in **b** in a larger scale.

The 22 symmetry-unique pMBA ligands were distinguished by using the TOCSY technique (see Fig. 3 and a full-page version in Supplementary Fig. 1). This collection method allowed for the determination of through-bond correlations and, therefore, enabled the separation of chemical shifts belonging to each pMBA ligand. HSQC gave correlations to which carbon the particular proton is attached, thus, identifying protons H_a_ and H_b_. In addition, from the HSQC spectra, multiple correlations for C_a_ and C_b_ carbons (a1, a2, b1 and b2) for rigid (as opposed to dynamic) ligands could be identified (Fig. 4 and Supplementary Fig. 2).

Combined, TOCSY and HSQC gave self-consistent information resolving the chemical shift shielding arising from the local chemical environment of each symmetry-equivalent pMBA ligand (Supplementary Table 1). Assignment of the chemical shift of each proton was based initially on the TOCSY spectrum. This enabled numbering of the ligands sequentially from downfield to upfield. The ligands are numbered thus pMBA-1, pMBA-2, …, where pMBA-1 is the most downfield ligand (Figs 1a and 2a)

**Figure 5:**
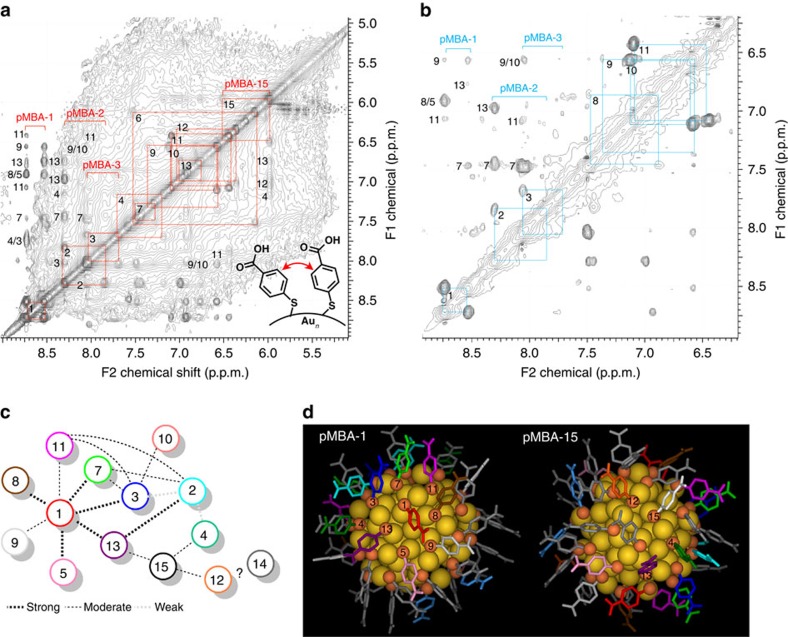
Through-space correlation data. (**a**) NOESY and (**b**) ROESY spectra of Au_102_(pMBA)_44_ cluster in 0.3 M D_2_O–NaOH showing the assignment (red and blue squares) of the through-space correlation peaks between neighbouring pMBA ligands. (**c**) Connectivity map of the neighbouring ligands based on the NOESY and ROESY correlations with strong, moderate and weak correlations highlighted accordingly. (**d**) The respective ligand positions in two areas of the surface, centred around ligands pMBA-1 and pMBA-15.

Through-space correlations were obtained with NOESY and ROESY experiments, which were complementary and afforded information about connectivity of neighbouring ligands within 5 Å proximity (Fig. 5 and Supplementary Figs 3 and 4).

### Assignment of ligand arrangement on the cluster surface

Initial assignment of each of the 22 symmetry independent pMBA ligands to their respective proton chemical shifts was accomplished by comparison of the experimental TOCSY and HSQC correlation peaks to DFT-calculated proton and carbon chemical shifts for each of the symmetry-equivalent pMBA ligand based on the crystal structure of Au_102_(pMBA)_44_ as a model (Fig. 2b and Supplementary Table 1). The dynamics of ligands in solution was taken into consideration by MD simulation studies, which revealed that the dynamical behaviour of the ligands was affected by interactions with the neighbouring ligands, the available space around the ligands and constraints set by the parent gold–thiolate unit. The final assignment of the 22 pMBA ligands was made based on special ligand environments (see below), neighbouring ligand interactions and ligand dynamics.

### Special ligand environments

From the calculated spectra, a set of five special ligand environments could be identified arising from shielding effects imposed by the local chemical environment (marked by horizontal bars in Fig. 2a). These five environments are, in order of deshielding: (1) ligand-to-gold interaction; (2) ligand-to-sulfur interaction; (3) isolated ligand; (4) face-to-face aromatic interaction; and (5) edge-to-face aromatic interaction. Ligand-to-gold interactions were the most deshielded and gave the highest downfield shifts (8.8–8.2 p.p.m.), whereas isolated ligand and ligand-to-sulfur interactions fell in the same region (7.6–7.1 p.p.m.) as free pMBA (7.40 and 7.23 p.p.m.). Face-to-face aromatic interactions were likewise more deshielded and shifted downfield (8.0–7.3 p.p.m.) in comparison with free pMBA, while edge-to-face aromatic interactions shifted the most upfield (6.6–5.9 p.p.m.). Large difference (Δ) between H_a_ and H_b_ shifts was observed for ligands that experienced both shielding and deshielding ligand environments. For example, combination of edge-to-face and face-to-face interactions yielded this large difference, marked by ‘large Δ p.p.m.' in Fig. 2a.

Correlating the experimental TOCSY assignments with the calculated chemical shifts revealed two pMBA ligands (pMBA-1 and -2) having the most downfield shifts, which were assigned as ligands in the model having distinct ligand-to-gold interactions (Fig. 1b, left). pMBA-1 is located at the five-fold symmetry axis of the gold core. A second special ligand environment was traced to face-to-face aromatic interactions of three ligands (pMBA-3, -7 and -11) assembling into a ‘stack of three' that gave downfield shifts both in the experimental and calculated data (Fig. 1b, middle). The different chemical environment imposed by the static *π*-stacking interactions was clearly seen as multiple strong HSQC correlations for C_a_ and C_b_ carbons. Owing to face-to-face aromatic interactions from both sides, ligand pMBA-7 in the middle of the stack showed two HSQC correlations for both C_a_ and C_b_ carbons (129.4/132.5 and 129.1/131.9 p.p.m.), whereas only the C_a_ carbons of ligands pMBA-3 (132.8 and 133.6 p.p.m.) and -11 (129.0 and 132.3 p.p.m.) at the top and bottom of the stack were affected (Fig. 4). The most upfield shift, on the other hand, was identified as an edge-to-face aromatic interaction (pMBA-15), which also correlated well with the calculated model (Fig. 1b, middle). The last special ligand environment was observed with a pMBA ligand (pMBA-6) that had both symmetry-equivalent ligands attached to the same RS-Au-SR unit having the largest Δ value (1.39 p.p.m.) between protons H_a_ and H_b_ that resulted from both edge-to-face and face-to-face aromatic interactions with the neighbouring ligands.

### Through-space interactions

Whereas chemical shift shielding afforded information about the nature of the ligand interactions, through-space correlations gave information to which neighbouring ligands these interactions are formed. NOESY and ROESY spectra therefore afforded a connectivity map of neighbouring ligands, which could be used together with the previous assignment based on the local ligand environment (Fig. 5). The strongest NOESY and ROESY correlations were associated with ligands pMBA-1 and -15, which also suggested lesser mobility of these ligands and the neighbouring ligands they were interacting with. Thus, pMBA-1 showed NOESY/ROESY correlations to ligands pMBA-4/-3, -7, -8/-5, -9, -11 and -13 (Fig. 4). Of these the symmetry-equivalent site was already established for pMBA-3, -7 and -11 corroborating the earlier assignment. pMBA-7 also showed ROESY correlations to pMBA-1, -2 and -3 confirming their proximity and agreement with the model. pMBA-11 likewise gave correlations to pMBA-1 and -2 that were in close ranges. It should be noted that pMBA-1 and -11 are attached to the same RS-Au-SR unit and are therefore likely to interact. The same holds for ligands pMBA-2 and -3.

The assignment of the remaining ligands surrounding pMBA-1 at the five-fold symmetry axis was based on the best possible agreement of the experimental and calculated chemical shifts. pMBA-13 was assigned first as having a distinct upfield shift in the experimental spectrum (6.98–6.76 p.p.m., Δ=0.20 p.p.m.), which was most likely due to an edge-to-face aromatic interaction to a ligand that was assigned as pMBA-4 showing up more downfield (7.70–7.18 p.p.m.). Both ligands pMBA-4 and -13 had a NOESY correlation to pMBA-2 and -15, which were in close distance and agreed well with the model. The order of ligands pMBA-5, -8 and -9 was made based on the subtle changes in their chemical shift shielding, since they all appeared at the ‘middle range' (7.62–6.56 p.p.m., Δ=0.5–0.8 p.p.m.) and their symmetry-equivalent site was determined based on the best agreement with the experimental and calculated data (Supplementary Table 1).

Ligand pMBA-3 gave NOESY/ROESY correlations to ligands pMBA-2, -7 and -11, whose proximity was already confirmed and established, but also to pMBA-9/-10. This interaction was assigned to pMBA-10 due to its closer distance and more probable interaction to pMBA-3 based on the model in comparison with pMBA-9.

**Figure 6:**
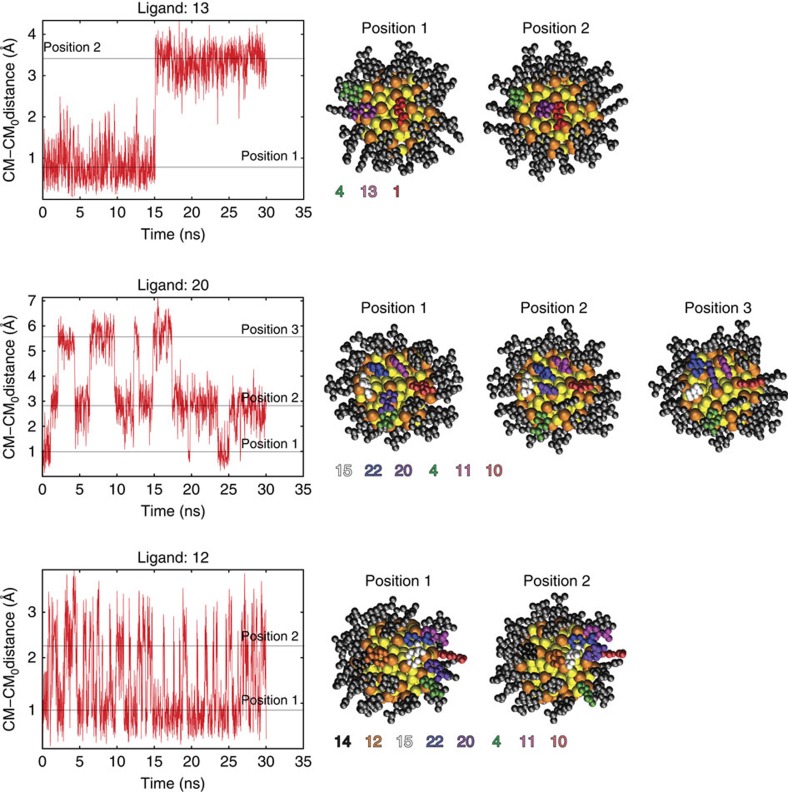
Dynamics of the centre of mass (CM) of selected ligands during the MD simulation. Distances are plotted with respect to the initial position of the CM. Structures visualize the differences in the orientation of the ligands at each position 1, 2 and 3. The colours of the ligands are as in Fig. 1.

Finally, the last through-space interactions were established for ligand pMBA-15 that showed NOESY correlations to ligands pMBA-4, -12 and -13. Of these, the assignment of pMBA-4 and -13 was already made. Therefore, the symmetry-equivalent site for pMBA-12 was assigned as the centre position of the long RS-Au-(SR)-Au-SR unit, also agreeing well with the experimental and calculated data (Fig. 1b, right). MD simulation studies showed this ligand to be one of the most dynamic ones, due to the flexibility of the long gold–thiolate unit (Figs 3b and 6 and Supplementary Table 2).

### Ligand dynamics

MD simulations were conducted to assess the different degrees of mobility and orientation dynamics of the ligands. The dynamics is visualized in Supplementary Movies 1 and 2 that visualize the dynamics of the particle over 30 ns in water. A more detailed analysis of the dynamics of three selected ligands are given in Fig. 6, Supplementary Table 2 and Supplementary Data 1. We observed that the extent of the chemical shift in the NMR spectra had a clear correlation to ligand mobility (Fig. 3). More static ligands gave stronger correlation peaks, whereas ligands that showed higher mobility in the MD simulation gave weaker correlation peaks (Supplementary Table 1). Strong TOCSY correlations were found for 15 pMBA ligands, of which 14 were assigned above. The remaining strong correlation (pMBA-14) in the upfield region of the proton spectrum (6.79–6.28 p.p.m.) was assigned based on its good agreement with the calculated data. The symmetry-equivalent site for pMBA-14 is a close neighbour to pMBA-12, which was established as one of the most mobile ligands being attached on the long gold–thiolate unit, and therefore does not show through-space correlation to pMBA-14, although correlation to the other closest neighbour pMBA-15 was observed (Fig. 1b, right; and Fig. 3).

The remaining weak TOCSY correlations were assigned by comparing the experimental chemical shifts with the calculated ones. These ligands also showed high mobility in the MD simulation studies. Ligands pMBA-16, -17 and -18 all gave similar TOCSY correlations in the downfield region with high Δ value (8.25–6.76 p.p.m., Δ=1.10–1.39 p.p.m.) indicating more than one type of interaction (Fig. 2). The observed downfield shifts can be explained by the interaction with the gold core that is very similar for all the three ligands. Ligand pMBA-19 appeared at the ‘middle range' (7.57–7.29 p.p.m., Δ=0.28 p.p.m.), whereas good agreement with the calculated data was found for ligands pMBA-20 and -21 in the upfield region of the spectrum (7.24–6.06 p.p.m., Δ=0.95–1.18 p.p.m.). This is reasonable since pMBA-19 is nearly isolated on the cluster surface while pMBA-20 and pMBA-21 both have edge-to-face and face-to-face characteristics. The last remaining ligand pMBA-22 was assigned to the symmetry-equivalent site at the other end of the long gold–thiolate unit.

## Discussion

We were able to interpret fully the very complicated ^1^H and ^13^C NMR spectra of the inorganic nanoparticle Au_102_(pMBA)_44_ in solution by using a combination of multidimensional NMR methods, DFT calculations and MD simulations. With help of DFT calculations and MD simulations we showed that the ligands have their own intrinsic orientation in close connection to the arrangement seen in the crystal structure. We also showed that different ligands can have considerably different dynamical behaviour, which is confirmed by both MD simulations and the observed signal strengths of the measured NMR shifts. We mapped the ligand–ligand interactions and ligand environments to the proton spectrum, which can be generalized to also other ligand-protected inorganic clusters and nanoparticles. Strong ligand–ligand interactions were seen to reduce dynamics of the ligands and cause splitting of the signal in HSQC, which meant that four signals could be measured for the carbon atoms of the phenyl ring that are bound to the hydrogen.

Our observations are important for future research of the ligated inorganic clusters in many ways. First, many of the known ligand-protected clusters have a well-defined molecular composition, including nanoparticles with perhaps 500 metal atoms and a well-developed plasmon, but lack empirical structure information regarding the ligand shell and ligand–metal interactions. NMR spectra for many of these clusters are reported, with severe congestion in the aromatic region of the spectra when the clusters are ligated by phenyl-containing ligands^14^,^16^,^25^. Our work gives ‘fingerprints' of typical proton NMR shifts expected for a number of different classes of weak ligand–ligand interactions, which should prove useful in future analyses of NMR data from clusters where the solid-state X-ray structure is not known. We propose that the multidimensional NMR measurements combined with DFT calculation of NMR spectra, augmented by traditional high-resolution electron microscopy, can now allow for empirical structural models of entire nanoparticles (including the ligand shell and ligand–metal interaction). Second, unsolved questions like reason for the different structural symmetry with different ligands could be answered using the methods used in this study. Finally, the results show that monitoring and controlling the ligand exchange of protected inorganic clusters could be possible with a single ligand accuracy using multidimensional NMR methods, allowing more rapid assessment of regio-isomerism than is presently possible. In the future this can lead to extremely highly controllable and accurate methods for functionalization of the ligated nanoparticles for various applications.

## Methods

### Sample preparation

Au_102_(pMBA)_44_ was synthesized as previously reported^15^. For NMR acquisition, a 1.0-ml saturated solution of Au_102_(pMBA)_44_was prepared in 0.3 M NaOH in D_2_O. The solution was centrifuged to remove insoluble Au_102_(pMBA)_44_.

### NMR spectroscopy

NMR data were acquired at 25 °C on a Varian Inova 500 MHz spectrometer equipped with three analytical channels and a 5-mm proton detect, triple resonance probe (HCN probe). Varian and Agilent softwares, VnmrJ2.2 and VnmrJ4.2, respectively, were used for spectrometer control and data processing. All pulse sequences were used without modifications made from the software release. The ^1^H-detected dimension was acquired as a downsampled 8-kHz free induction decay (FID) in quadrature mode with hardware digital signal processing from a 500-kHz oversampled data stream. The ^1^H *π*/2 pulse width was 7.5 μs. 1D ^1^H data were acquired with a *π*/4 pulse, 2.0 s acquisition time, 16 K complex points and 7.0 s recycle time. In the case of the 2D data acquisitions, the ^1^H directly detected dimension had a 0.25 s acquisition time, 2 K complex points and a 2.0 s recycle time. Some spectra were acquired with the residual water signal pre-saturated by a 1.5-s, 25-Hz continuous wave pulse centred on the residual water signal. The first two acquired data points were linear back-predicted. The 2D, indirectly detected dimension was modulated in phase-sensitive mode by using States-TPPI scheduling. In the case of the ^1^H–^1^H homonuclear NMR experiments, the indirectly acquired dimension was 8 kHz in 200 complex points. In the case of the ^1^H–^13^C{^13^C} HSQC, the ^13^C indirectly acquired dimension was 12.5 kHz in 128 complex points. In all 2D data sets, the interferogram was extended by a factor of two through linear forward prediction, weighted with a time-shifted Gaussian function for resolution enhancement and then the full, 2D data sets were zero-filled and Fourier-transformed. The homonuclear experiments had a final matrix size of 2 × 2 K complex points and 4 Hz per point, and the HSQC was 2 K × 512 complex points and 4 and 24 Hz per point, respectively. The HSQC data were acquired by taking 256 signal averaging transients per increment. TOCSY data were acquired by using 0.024 and 0.0640-s 8-kHz DIPSI-2 isotropic mixing sequences and taking 32 signal averaging transients per increment. The TOCSY with 0.064-s mixing was used in our results. NOESY data were acquired by providing 0.080, 0.150 and 0.200 s dipolar contact time and taking 64 signal averaging transients per increment. The nuclear Overhauser effect build-up was examined qualitatively to maximize cross-peak intensity without interference from spin diffusion. The 0.200-s mixing period provided the best cross-peak intensity for simple distance constraints in our results.

### Computational methods

The NMR shifts were calculated for a theoretically relaxed structure obtained from the solved crystal structure of Au_102_(pMBA)_44_ (ref. 9). The structure was relaxed by using DFT as implemented in code-package GPAW^26^ that uses a real-space grid with projector-augmented waves (PAW). The electron–electron interactions were treated by using the Perdew–Burke–Ernzerhof (PBE)^27^ exchange-correlation functional, and the wave functions and electron density were described in a real-space grid of 0.2 Å grid spacing. The relaxation of the atomic structure was stopped when the residual forces on atoms were below 0.1 eV Å^−1^.

NMR shifts were calculated using Gauge-Including Projector-Augmented Wave (GIPAW) formalism as implemented in pseudopotential- and planewave-based DFT code-package Quantum Espresso^28^. We used ultrasoft PBE-PAW pseudopotentials (http://www.quantum-espresso.org/pseudopotentials/), a kinetic energy cutoff of 950 eV for wave functions and 9,520 eV for the charge density and potential. The energy convergence criterion for the self-consistency was set to 1.4·10^−11^ eV. Total chemical shifts were defined as *σ*=*σ*(ref)−*σ*(calc), where *σ*(ref) was fixed to 31.099 p.p.m. for ^1^H shifts and for 166.049 p.p.m. for ^13^C shifts. Both of the selected reference values give a total average of all the calculated ^1^H and ^13^C shifts that is comparable to the experimental results. From the calculated NMR shifts we mainly concentrate on the symmetrically averaged values of 22 unique pMBA ligands as seen also in the experiment. A couple of clear overestimations in the shifts for the specific individual atoms of the ligands were found, most probably caused by the unique ligand arrangement and ligand–ligand interactions that are static in NMR calculations but not in the experiment.

Dynamics of the ligands was studied by classical MD simulations in a water environment for 30 ns time at room temperature using the GROMACS^29^ package. To describe the Au_102_(pMBA)_44_ cluster, we used an AMBER-compatible molecular mechanics force field for thiolate-protected gold clusters that has been recently developed and tested in our group (Pohjolainen *et al*, submitted manuscript). The principles of the force field were as follows: (1) no constraints included in the gold core, with the atoms interacting via a simple pair potential (Lennard–Jones type); (2) for transferability, the charges of gold atoms were set to zero; (3) for the Au-S interface, equilibrium bond lengths and angles were averaged from our previous DFT calculations for the Au_102_(pMBA)_44_ structure, and force constants were adopted from a previous work^30^; and (4) for pMBA parameters, atom types were chosen from existing AMBER atom types, and partial charges were calculated from RESP fit.

Dynamical behaviour of the 22 symmetrically unique ligands were estimated from the MD trajectory both visually and by calculating an averaged root mean squared deviations for all the ligands separately, in which we concentrated on the atoms that were relevant for the measured NMR shifts. The reference structure in root mean squared deviation calculations were taken after initial thermalization but before any conformational changes were seen in the ligand layer.

## Additional information

**How to cite this article:** Salorinne, K. *et al.* Conformation and dynamics of the ligand shell of a water-soluble Au_102_ nanoparticle. *Nat. Commun.* 7:10401 doi: 10.1038/ncomms10401 (2016).

## Supplementary Material

Supplementary Figures and Supplementary Tables Supplementary Figures 1-4 and Supplementary Tables 1-2

Supplementary Movie 1 The movie visualizes about 30 ns MD simulation of Au102(pMBA)44 in a 5nm3 cubic box filled with water molecules. In the visualization water is not shown. The simulation was done by using Gromacs version 4.5. The leap-frog Verlet integrator was used with time step of 2 fs. Other parameters are: a 1.0 nm Lennard-Jones cut-off with dispersion correction for energy and pressure, PME electrostatics with a 1.0 nm cut-off and 0.12 nm grid spacing, the velocityrescale thermostat with a reference temperature of 300 K and coupling time constant of 0.1 ps, and the Berendsen barostat with a reference pressure of 1 bar and coupling time constant of 1 ps. All bond lengths were constrained with the LINCS algorithm and no constraint was used for gold core. The movies contain 750 frames and the time between two frames is 40 ps. The colors of ligands are same as in Figure 6 in the main text. The coordinates of the full MD trajectory are given in Supplementary Data 1.

Supplementary Movie 2 Same visualization as Supplementary Movie 1 but from a different viewing direction.

Supplementary Data 1 Coordinates of the full MD trajectory.
