# Supplementary material for: Conformation and dynamics of the ligand shell of a water-soluble Au102 nanoparticle
Source: Nat Commun. 2016 Jan 21;7:10401. doi: 10.1038/ncomms10401 (PMC4736050; doi:10.1038/ncomms10401)
Supplement: Supplementary Figures and Supplementary Tables — Supplementary Figures 1-4 and Supplementary Tables 1-2 [file ncomms10401-s1.pdf]

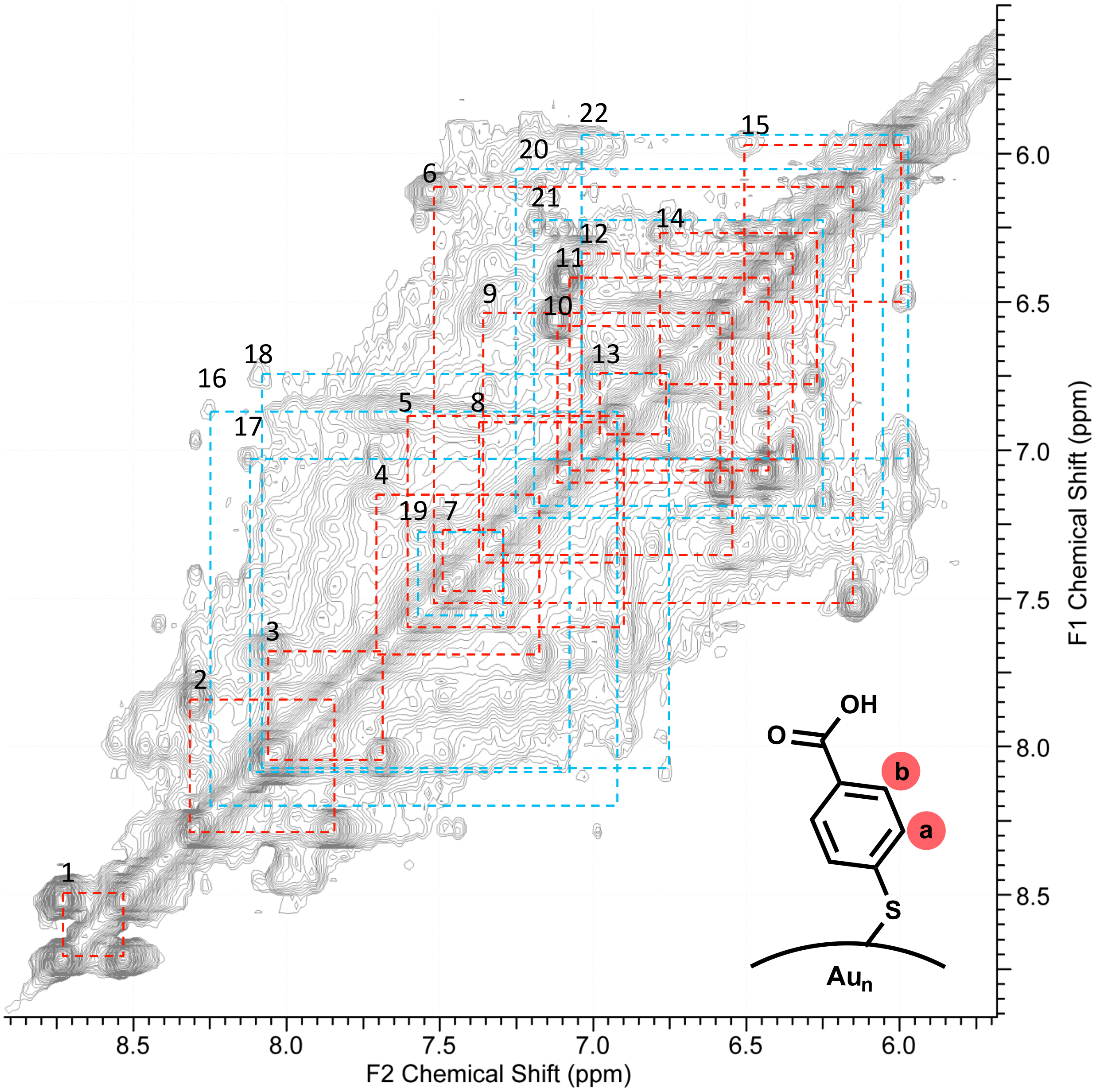

Supplementary Figure 1. Full page TOCSY spectrum of $Au_{102}(pMBA)_{44}$ in 0.3M NaOD-D_2O at 600 MHz. Chemical shift assignments for each pMBA ligand are shown with red (strong correlations) and blue (weak correlations) dotted squares.

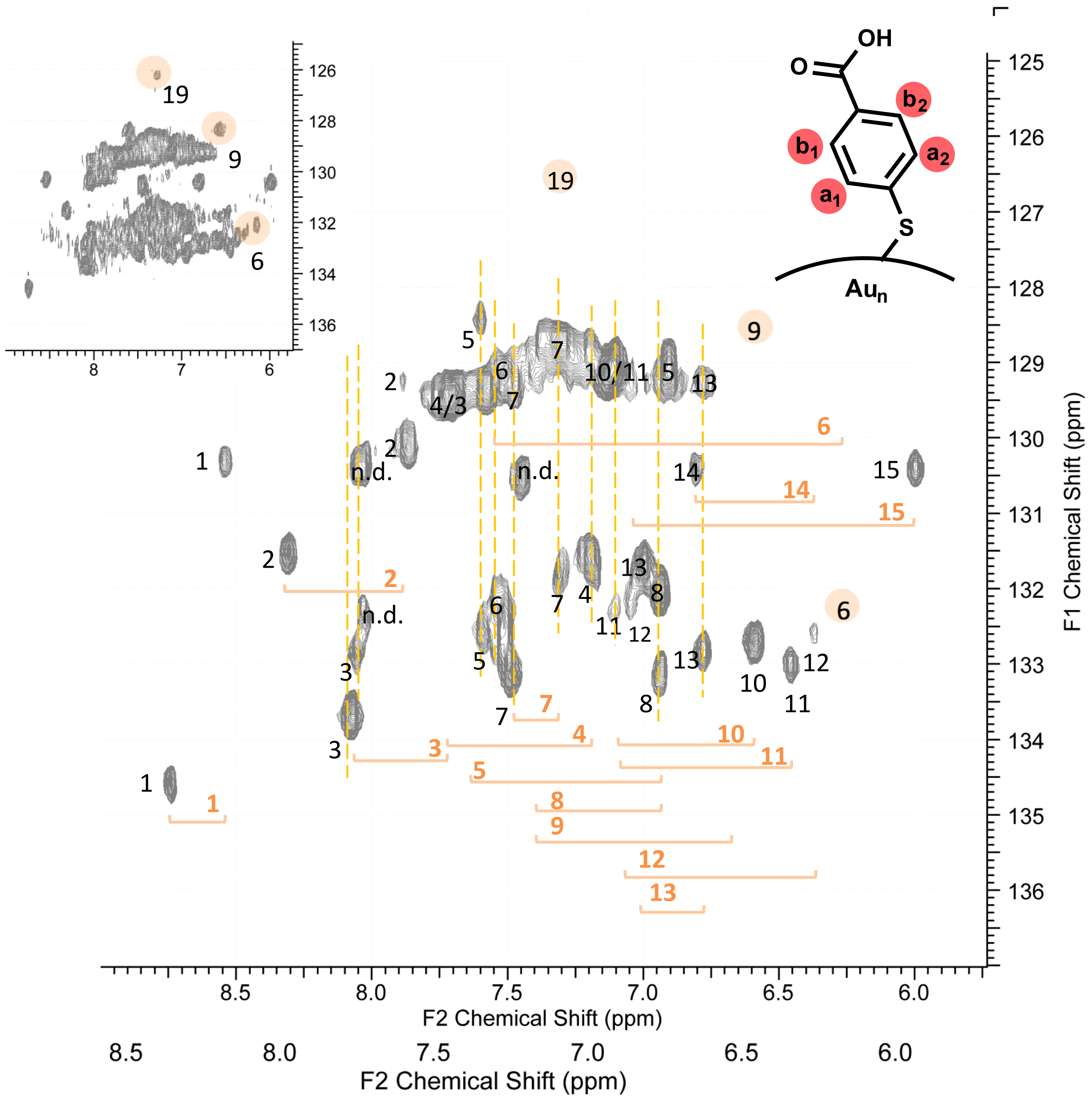

Supplementary Figure 2. Full page HSQC spectrum of $Au_{102}(pMBA)_{44}$ in 0.3M NaOD-D_2O at 600 MHz. Chemical shift assignments of pMBA ligands are shown with numbers. Orange bars indicate the chemical shifts belonging to the same pMBA ligand.

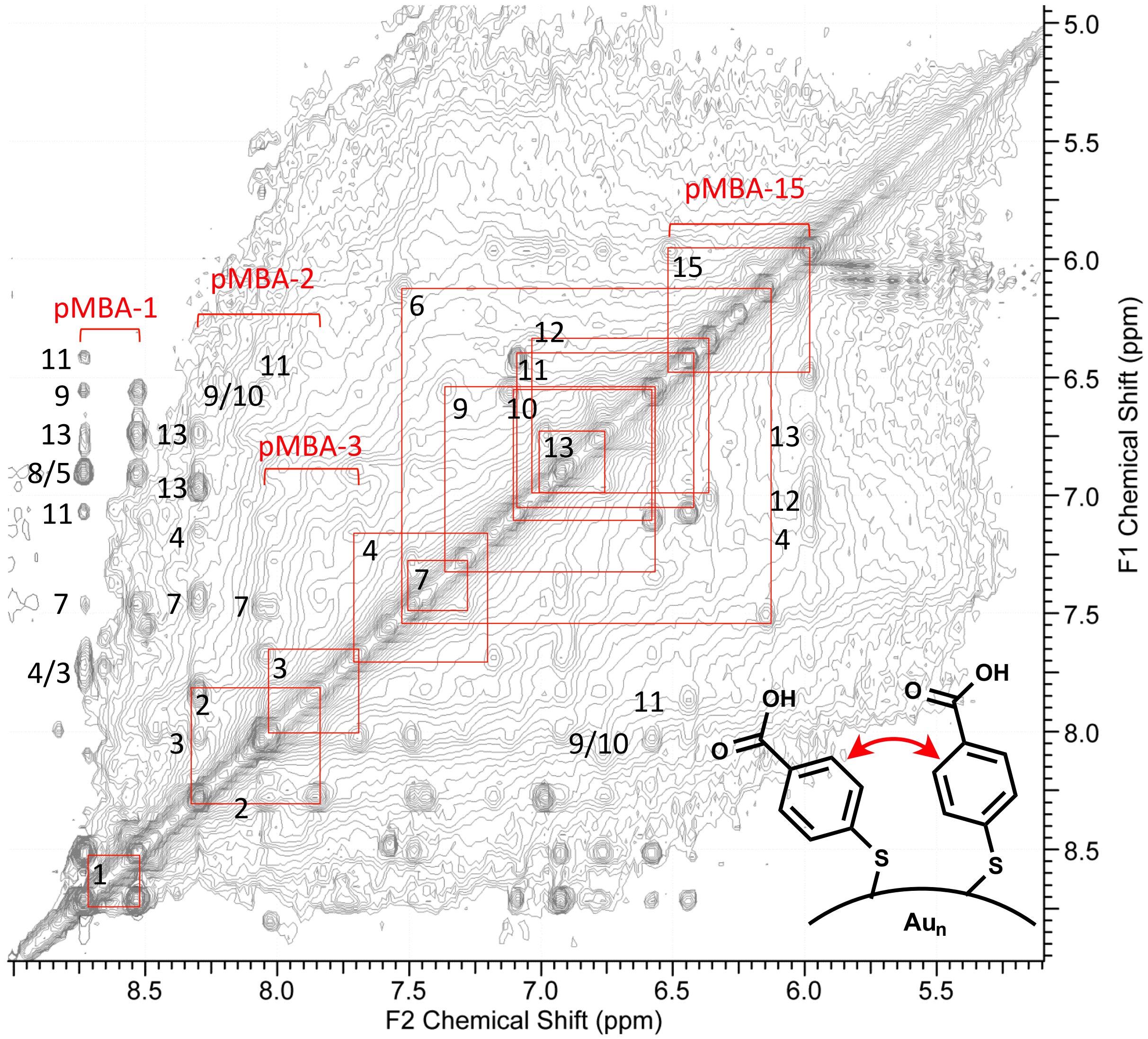

Supplementary Figure 3. Full page NOESY spectrum of $Au_{102}(pMBA)_{44}$ in 0.3M NaOD-D_2O at 600 MHz. Through space correlations are marked with red squares.

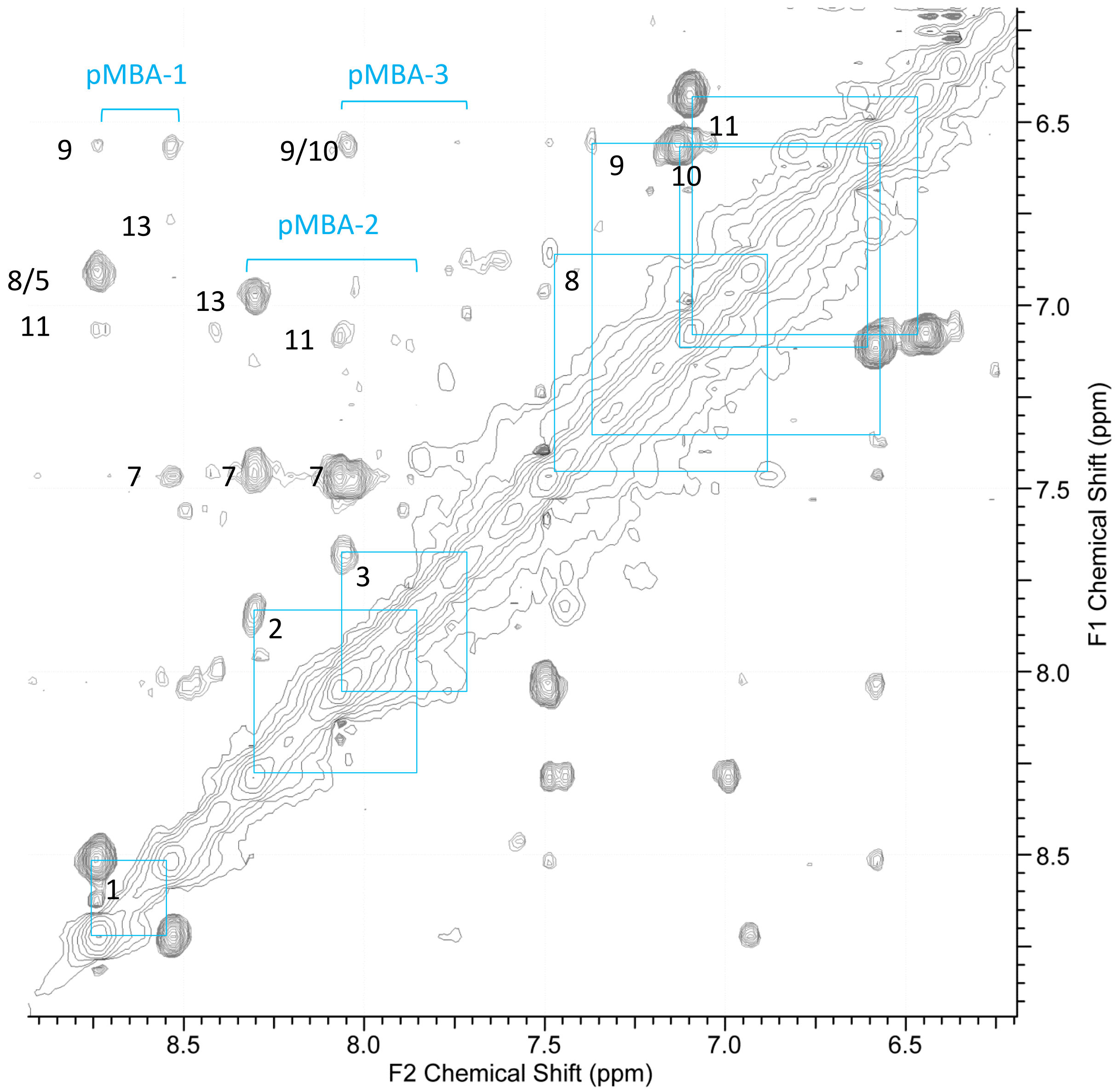

Supplementary Figure 4. Full page ROESY spectrum of $Au_{102}(pMBA)_{44}$ in 0.3M NaOD-D_2O at 600 MHz. Through space correlations are marked with blue squares.

Supplementary Table 1. ^{1}H NMR chemical shift assignment based on TOCSY and HSQC correlations and DFT calculations.

TOCSY (ppm)	D[1] (ppm)	HSQC (ppm)	pMBA[2]	Calculated[3] ^{1}H (ppm)	Calculated[3] ^{13}C (ppm)	H_a and H_b assignment[4]	Dynamics[5]
8.73	0.20	134.6	**1** (S3)	9.77<br>(9.63, 9.90)	137.83<br>(141.55, 134.11)	1b	0.8
8.53		130.3		9.04<br>(8.43, 9.65)	131.54<br>(126.30, 136.78)	1a	s
8.30	0.45	131.7	**2** (S22)	9.13<br>(9.97, 8.28)	130.95<br>(131.47, 130.42)	2b	1.0
7.85		129.7, 129.2 (w)		8.62<br>(8.36, 8.87)	133.86<br>(129.02, 138.70)	2a	s
8.05	0.36	133.6, 132.8	**3** (S8)	8.22<br>(8.22, 22.89)	131.05<br>(131.05, 188.06)	3b	0.6
7.69		129.4		6.84<br>(5.57, 8.10)	131.78<br>(129.54, 134.03)	3a	s
7.70	0.52	129.4	**4** (S11)	8.36<br>(7.65, 9.07)	132.37<br>(129.31, 135.43)	4a	2.2*
7.18		131.9		7.86<br>(7.91, 7.80)	129.42<br>(126.33, 132.50)	4b	s/m
7.62	0.72	130.0, 132.5	**5** (S1)	8.28<br>(8.10, 8.45)	133.76<br>(136.73, 130.80)	5a	0.9
6.90		129.2		7.89<br>(7.65 8.12)	130.23<br>(132.72, 127.74)	5b	s
7.53	1.39	132.7, 129.2	**6** (S16)	8.07<br>(11.14, 8.07)	133.23<br>(68.09, 133.23)	6a	2.5
6.14		132.0		5.73<br>(5.52, 5.93)	131.50<br>(152.98, 110.01)	6b	m/vm
7.49	0.20	132.5, 129.4	**7** (S13)	7.19<br>(7.14, 7.24)	129.98<br>(132.35, 127.60)	7a	0.7
7.29		131.9, 129.1		7.10<br>(19.10, 7.10)	129.52<br>(170.38, 129.52)	7b	s
7.38	0.46	128.8 (w)	**8** (S4)	7.79<br>(7.31, 8.28)	135.89<br>(137.38, 134.40)	8a	1.9
6.92		131.9, 133.1		7.40<br>(6.38, 8.42)	130.80<br>(127.86, 132.38)	8b	m
7.36	0.77	128.8 (w)	**9** (S5)	7.51<br>(8.67, 6.34)	132.50<br>(135.17, 129.83)	9b	1.3
6.56		128.3		6.83<br>(7.60, 6.05)	132.17<br>(129.47, 134.87)	9a	s
7.12	0.54	129.1	**10** (S20)	7.57<br>(8.69, 6.45)	128.87<br>(122.86, 134.88)	10a	0.6
6.58		132.5		7.02<br>(8.39, 5.65)	132.85<br>(136.03, 129.67)	10b	s
7.09	0.65	132.3, 129.0	**11** (S9)	7.15<br>(6.09, 8.21)	131.66<br>(127.91, 135.40)	11a	0.6

6.44		132.9		6.94<br>(7.74, 6.15)	129.96<br>(128.60, 131.31)	11b	s
7.03	0.67	132.2	**12** (S21)	7.52<br>(7.30, 7.73)	108.65<br>(96.29, 121.00)	12b	2.8**
6.36		132.4		5.69<br>(4.66, 5.69)	124.16<br>(94.78, 124.16)	12a	m/vm
6.98	0.22	132.0	**13** (S2)	7.64<br>(7.29, 7.99)	130.19<br>(133.50, 126.87)	13a	2.3*
6.76		132.9, 129.3		7.44<br>(7.44, -10.22)	128.41<br>(128.41, 259.46)	13b	s/m
6.79	0.51	130.1	**14** (S17)	6.98<br>(6.69, 7.29)	133.00<br>(133.38, 132.61)	14a	2.0
6.28		n.d.		6.92<br>(6.13, 7.71)	128.77<br>(126.34, 131.19)	14b	m
6.52	0.53	n.d.	**15** (S18)	6.59<br>(6.13, 7.04)	137.92<br>(141.82, 134.01)	15a	1.6
5.99		130.4		4.67<br>(3.29, 6.04)	83.75<br>(75.94, 91.55)	15b	m
8.25	1.39	n.d.	**16** (S6)	8.31<br>(7.76, 8.85)	129.68<br>(128.96, 130.40)	16b	2.5
6.86		129.8 (w)		7.69<br>(7.03, 8.35)	133.86<br>(131.42, 136.30)	16a	m/vm
8.13	1.10	n.d.	**17** (S12)	8.21<br>(8.13, 8.28)	129.61<br>(131.60, 127.61)	17b	1.4
7.03		n.d.		7.98<br>(7.47, 8.48)	134.68<br>(130.19, 139.16)	17a	s
8.10	1.34	n.d.	**18** (S10)	8.17<br>(8.06, 8.28)	129.05<br>(129.13, 128.96)	18b	2.4
6.76		n.d.		7.70<br>(7.09, 8.30)	133.34<br>(131.07, 133.84)	18a	m
7.57	0.28	129.2	**19** (S7)	8.19<br>(7.59, 8.79)	134.38<br>(135.83, 132.94)	19a	2.6*
7.29		126.3		7.58<br>(7.49, 7.67)	123.67<br>(126.51,120.82)	19b	m/vm
7.24	1.18	128.8 (w)	**20** (S19)	7.18<br>(6.88, 7.48)	114.93<br>(104.04, 125.82)	20b	3.9
6.06		n.d.		6.16<br>(5.10, 7.23)	132.76<br>(126.62, 138.91)	20a	vm
7.19	0.95	128.9 (w)	**21** (S15)	7.54<br>(7.42, 7.66)	129.59<br>(128.30, 130.88)	21b	2.3
6.24		n.d.		6.59<br>(5.59, 7.60)	130.63<br>(126.63, 134.63)	21a	m
7.04	1.06	n.d.	**22** (S14)	7.47<br>(7.19, 7.74)	133.94<br>(130.10, 137.77)	22a	1.7
5.98		n.d.		7.45<br>(8.22, 6.67)	130.12<br>(127.86, 132.38)	22b	m

n.d. = not determined. [1] Δ ppm of H_a and H_b TOCSY chemical shifts. [2] Assignment of pMBA ligand. Parenthesis shows the number of the sulfur atom of the respective pMBA ligand in the crystal structure. [3] Calculated chemical shifts are given as averages for the symmetrically equivalent hydrogen and carbon atoms a1, a2, b1 and b2 in parenthesis. The overall averages of a and b type of hydrogen and carbon atoms are given without parenthesis. There are some non-realistic overestimations in the results that are neglected in calculations of the overall averages. These correspond to the ligands 3b, 6a, 7a, 12a and 13b. These overestimations are reasonable to be omitted due to the dynamical effects present in experiment that average out the extreme static arrangement of some very strongly interacting ligands in calculation. [4] Ha and Hb assignment separates a and b type of carbon and hydrogen atoms first based on experimental results and second based on calculated results. The assignment of Ha and Hb protons are taken from calculations. For each ligand the proton with the most downfield chemical shift is given first in the table, for both experimental and calculated results. [5] The root mean square displacements (rsmd) of the ligands are calculated from MD simulation and are averaged over Ha, Hb, Ca and Cb atoms of each of the 22 ligands. Rsmd is given in Ångstroms. Ligands have been cathegorized to three groups: s = static (rmsd < 1.5), m = mobile ($1.5 <$ rmsd < 2.5) and vm = very mobile (rmsd < 2.5); * pMBA-13 has two favorable positions and during MD-simulation it stays for long time stacked with pMBA-1 that is different than the reference initial orientation. This overestimated the dynamics of pMBA-13. pMBA-4 and pMBA-19 are the neighboring of pMBA-13 and are also affected by the orientation of pMBA-13. ** pMBA-12 is not originally stacked with pMBA-15 but will be during the simulation for considerable time of the full simulation. Dynamics of pMBA-12 is probably less than the calculated parameter is.

Supplementary Table 2. The relative time in percentages for the orientations that each of the ligands spend during the MD simulation. Ligands that show no remarkable change in their position during the MD simulation are omitted. Percentages are estimated based on the behavior of the center of the mass of each ligand during the MD-simulation.

Ligand	Position 1	Position 2	Position 3
13	59.6 %	40.4 %	-
20	13.9 %	55.6 %	30.5 %
19	66.2 %	23.4 %	10.4 %
22	67.2 %	32.8 %	-
12	42.3 %	57.7 %	-
18	52.6 %	47.4 %	-
8	56.3 %	43.7 %	-
9	76.6 %	23.4 %	-
15	65.5 %	34.5 %	-
14	81.2 %	18.8 %	-
